# The pattern of a specimen of *Pycnogonum litorale* (Arthropoda, Pycnogonida) with a supernumerary leg can be explained with the “boundary model” of appendage formation

**DOI:** 10.1007/s00114-016-1333-8

**Published:** 2016-01-30

**Authors:** Gerhard Scholtz, Georg Brenneis

**Affiliations:** Humboldt-Universität zu Berlin, Institut für Biologie, Vergleichende Zoologie, Philippstr. 13, 10115 Berlin, Germany; Neuroscience Program, Wellesley College, 106 Central Street, Wellesley, MA 02481 USA

**Keywords:** Malformation, Regeneration, Limb development, Morphology, Micro-computed tomography, Sea spider, Pantopoda

## Abstract

A malformed adult female specimen of *Pycnogonum litorale* (Pycnogonida) with a supernumerary leg in the right body half is described concerning external and internal structures. The specimen was maintained in our laboratory culture after an injury in the right trunk region during a late postembryonic stage. The supernumerary leg is located between the second and third walking legs. The lateral processes connecting to these walking legs are fused to one large structure. Likewise, the coxae 1 of the second and third walking legs and of the supernumerary leg are fused to different degrees. The supernumerary leg is a complete walking leg with mirror image symmetry as evidenced by the position of joints and muscles. It is slightly smaller than the normal legs, but internally, it contains a branch of the ovary and a gut diverticulum as the other legs. The causes for this malformation pattern found in the *Pycnogonum* individual are reconstructed in the light of extirpation experiments in insects, which led to supernumerary mirror image legs, and the “boundary model” for appendage differentiation.

## Introduction

Animal malformations, or teratologies, and unusual morphologies have always attracted human attention. This is reflected in numerous specimens housed in curiosity cabinets and natural history collections. Yet, since Geoffroy Saint-Hilaire’s (1826) pioneer work and, in particular, the groundbreaking study of Bateson (1894) on the categorization and importance of animal malformations and irregularities, these naturally occurring or experimentally produced deviations from the normal patterns of body organizations played a crucial role for the understanding of developmental mechanisms and evolutionary processes (see Blumberg 2009). In a recent article, Guinard even proposed an “evolutionary teratology” as a discipline in its own right that “highlights the production of developmental anomalies (more or less drastic) over evolutionary times, which become integral parts of groups and taxa” (Guinard 2015, p. 20).

There are a few reported cases of malformations in the marine Pycnogonida, also known as Pantopoda or sea spiders, an arthropod group that is nowadays most convincingly placed within the Chelicerata (for discussion, see Giribet and Edgecombe 2013). Most of these instances are of anecdotal character (Dogiel 1911; Schimkewitsch and Dogiel 1913; Bouvier 1914; Arita 1936; Ohshima 1942a, b; Gordon 1932; Hedgpeth 1947; Child 1979; Stock 1987) and the described structural abnormalities concern the duplication, fusion, or absence of limbs and other body parts and have been most likely caused by irregular regeneration processes after injury of the animals. Furthermore, exceptional cases of gynandromorphy have been documented in the otherwise strictly diecious and mostly sexually dimorphic animals (Child and Nakamura 1982; Krapp and Viquez 2011; Lucena et al. 2015). In general, the observed patterns of these malformations correspond to those found in other arthropods (Bateson 1894). However, compared with the remaining chelicerate groups (e.g., Patten 1896; Brauer 1917; Ćurčić et al. 1991; Jacuński et al. 2005; David 2012) as well as with crustaceans (e.g., Bateson 1894; Shelton et al. 1981; Scholtz 2014; Scholtz et al. 2014), myriapods (e.g., Hubert 1968; Juberthie-Jupeau 1961; Leśniewska et al. 2009; Janssen 2013), and hexapods (e.g., Bateson 1894; Cappe de Baillon 1927; Cockayne 1929; Balazuc 1948, 1958; Puissegur and Benadona 1973; Hesse-Honegger 1998), well-documented instances of pycnogonid malformations remain scarce. Presumably, this is rather due to the lower number of pycnogonid investigations than to a lack of regeneration abilities or a lower frequency of cases.

In fact, the reported examples of pycnogonid malformations indicate a high potential of wound healing and regeneration in these animals. Yet, in most cases, the actual causes of the specific structural abnormalities are unknown, and the few experiments that have been conducted are somewhat ambiguous and contradictory in their interpretation (Loeb 1895; Morgan 1904). Studies in other chelicerates have shown that malformations are not only caused by regeneration but also by exposure to heavy metals (Itow et al. 1998), X-rays (Seitz 1966), increased temperature (Napiórkowska et al. 2015), and the misexpression of genes (Sharma et al. 2015). Hence, similar mechanism can be expected to act with respect to pycnogonid malformations, in addition to irregular regeneration.

Here, we describe an adult female specimen of *Pycnogonum litorale* Strøm, 1762 with a supernumerary limb, which is situated between the right second and third walking legs. This individual was unintentionally damaged during its first juvenile stage (=sixth postembryonic instar) in the trunk region between the right second and third walking legs. For the first time, we use micro-computed tomography (micro-CT) to study not only the external morphology but also the internal organization of a malformed pycnogonid. Except for its mirror image organization and a somewhat deviating pattern of gut diverticula, the supernumerary limb shows a high degree of external and internal similarity to the other walking legs.

The pattern of the observed malformation is discussed in the light of extirpation experiments in insects, where the formation of additional legs could be induced (Bohn 1974), and with respect to the “boundary model” for insect appendages (Meinhardt 1986, 2008).

## Material and methods

### Preparation, fixation, and photography

The specimen of *P. litorale* is an adult female with a body length of 11 mm measured from the tip of the proboscis to the end of the anal tubercle. It was raised in our laboratory culture of this species (for details, see Ungerer and Scholtz 2009). The living animal was anaesthetized with CO_2_ and photographed from dorsal and ventral perspectives with a Keyence VHX-1000 microscope by combining stacks of images at various *z*-levels with the implemented software. Afterwards, the animal was fixed in Bouin’s solution (saturated aqueous picric acid, pure acetic acid, and 10 % formaldehyde solution) for several hours at room temperature and subsequently washed and stored in 70 % ethanol. Additional pictures of the fixed specimen were taken with a Zeiss Lumar V12 stereomicroscope equipped with epifluorescence, making use of the green autofluorescence of the animal’s cuticle when excited with blue light.

### Micro-computed tomography

The sample was dehydrated with a graded ethanol series and incubated in a 1 % iodine solution (iodine, resublimated [Carl Roth GmbH & Co. KG, Karlsruhe, Germany; cat. #X864.1] in 99.8 % ethanol) overnight. After incubation, the sample was washed several times in 99.8 % ethanol and transferred into a vial. Scans were performed with an Xradia MicroXCT-200 Xray imaging system (Carl Zeiss Microscopy GmbH). Settings were optimized for the specimen, and objectives were chosen according to sample size and region of interest. Accordingly, the 0.39× and 4× objectives were used resulting in pixel sizes of 34.76 and 5.53 μm/px, respectively. Samples were scanned in 99.8 % ethanol. Exposure times were 2 s (0.39× scan) and 15 s (4× scan). Scanning parameters were 30 kV and 6 W, resulting in a current of 200 μA. Tomography projections were reconstructed by using the XMReconstructor software (Carl Zeiss Microscopy GmbH), resulting in images stacks (TIFF format). All scans were performed by using Binning 2 (summarizing 4 px, resulting in noise reduction) and subsequently reconstructed by using Binning 1 (full resolution) to avoid information loss.

Analyses of the micro-CT data were performed with the 3D reconstruction program “Imaris” (Bitplane AG, Switzerland, version 7.0.0). Within the “surpass mode” of this program, 3D volumes are generated from the recorded image stacks. Volumes are shown either in the default “maximum intensity projection” (MIP) or alternatively in the “Blend” option, which renders scanned structures non-transparent and thus facilitates evaluation of the external shape of an object. To virtually remove “non-target” regions that obstruct the view of more interiorly located substructures of interest, “clipping planes” were applied. “Oblique slicers” were used to create virtual sections with specific orientation through different regions of the specimen. All midgut components included in the 4× scan were manually segmented using the “surface tool” and upon masking of all surrounding structures added as additional channel into the 4× scan. This enabled to better illustrate the position, course, and branching pattern of the midgut diverticula within the specimen.

### Data presentation

Global contrast and brightness values of some of the images were adjusted using Adobe Photoshop CS3. Figures were compiled in Adobe Illustrator CS3.

## Results

### History of the individual

Like the majority of pycnogonids, *P. litorale* hatches as a minute protonymphon larva with only three leg-bearing segments. During anamorphic postembryonic development, which encompasses six additional instars up to the juvenile, the more posterior segments and corresponding walking legs are sequentially added (Vilpoux and Waloszek 2003).

During spring 2013, the studied individual of *P. litorale* was transferred to a separate tank. At that time, it was in the sixth instar stage with a still undeveloped fourth (last) walking leg pair (see Vilpoux and Waloszek 2003). In this stage, the cuticle is still very soft and the animal was during transfer unintentionally damaged in the trunk region between the second and third walking legs. Yet, the animal survived, and after several months, it was found in the state that is described in the following.

### Description of the malformed individual

#### External organization

**Fig. 1:**
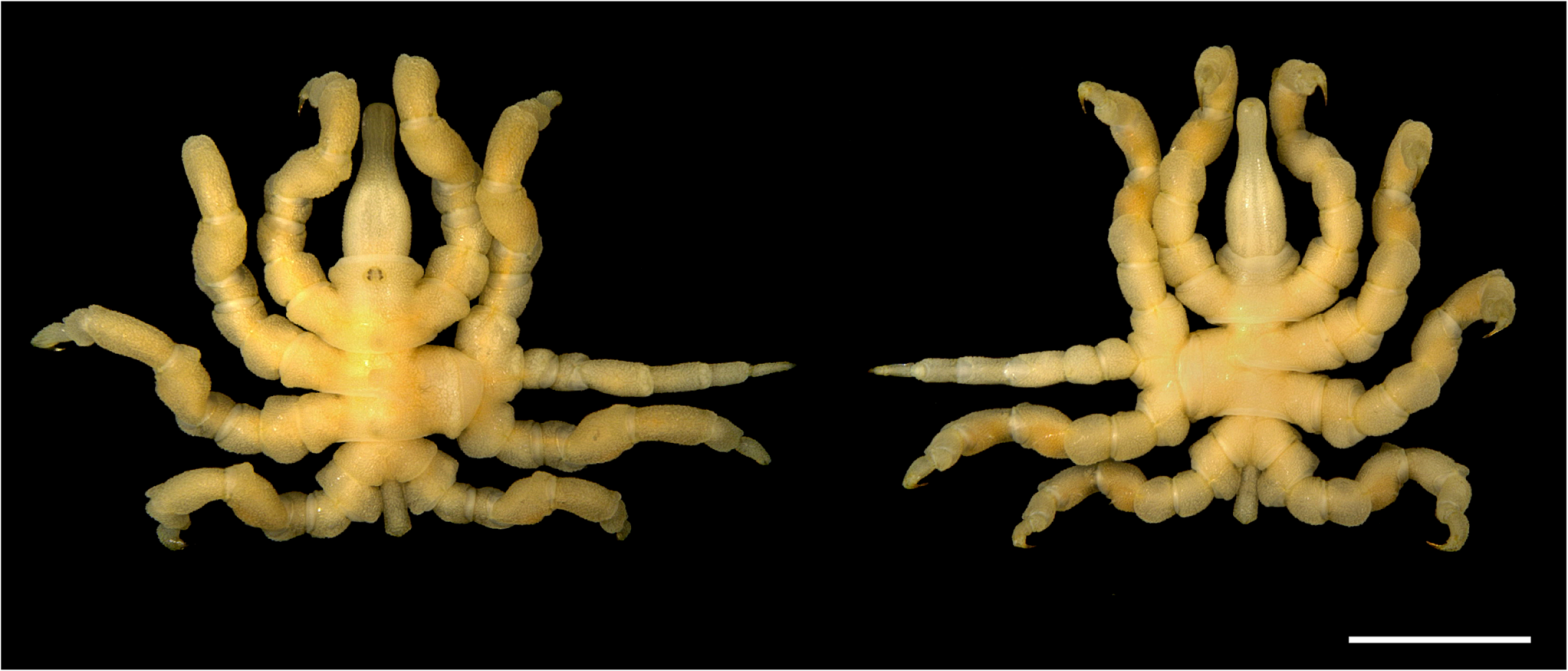
Living adult *Pycnogonum litorale* female with malformed right body half showing some fusions and a supernumerary leg. Anterior to the top. *Scale bar* 5 mm. *Left side*: dorsal aspect. *Right side*: ventral view

**Fig. 2:**
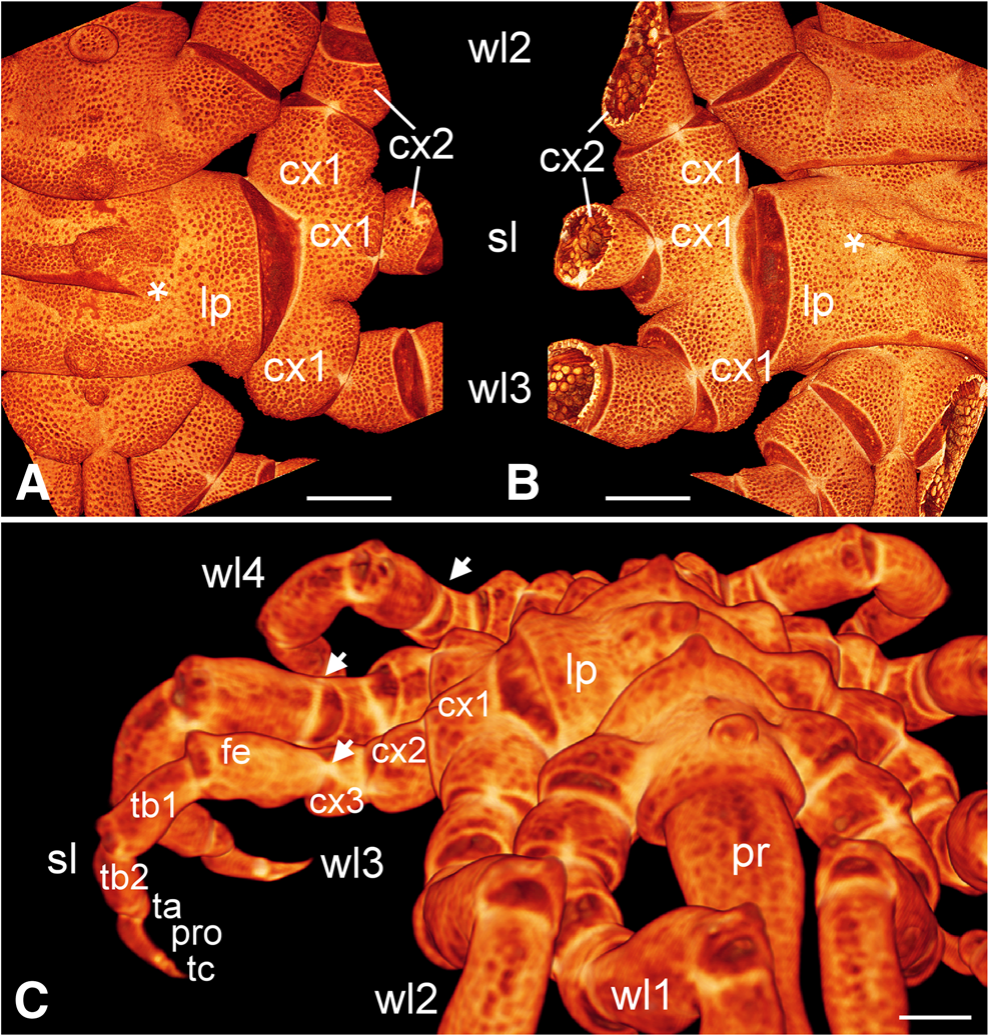
Details of the malformed right trunk region (**a**, **b**) and the posture of the supernumerary leg (**c**). Micro-CT scans, reconstructed with Imaris 3D software. In **a**, **b** anterior to the top, **c** shows a frontal perspective. *Scale bars* 1 mm. **a** Dorsal view of the right trunk. The malformations of the right body half in the area of the second and third walking leg segments are shown: the distorted central region (*asterisk*), the fused lateral processes (*lp*), as well as the fusion of the coxae 1 (*cx1*) of the second and third walking legs (*wl2*, *wl3*) and of the supernumerary leg (*sl*). **b** Ventral view of the right trunk showing the malformations of the ventral side, in particular, the fused lateral processes (*lp*) and the oblique segmental boundary between the second and third walking leg segments (*asterisk*). **c** Frontal view of a 3D reconstruction of the specimen depicting the normal posture and podomere number (coxae 1 to 3 (*cx1*, *cx2*, *cx3*), femur (*fe*), tibiae 1 and 2 (*tb1*, *tb2*), tarsus (*ta*), propodus (*pro*), and terminal claw (*tc*)), but the mirror image orientation as indicated by the position of joints (*arrows*, see Fig. 4a, b) of the supernumerary leg (*sl*) when compared with the other walking legs (*wl1–wl4*). *lp* fused lateral processes, *pr* proboscis

**Fig. 3:**
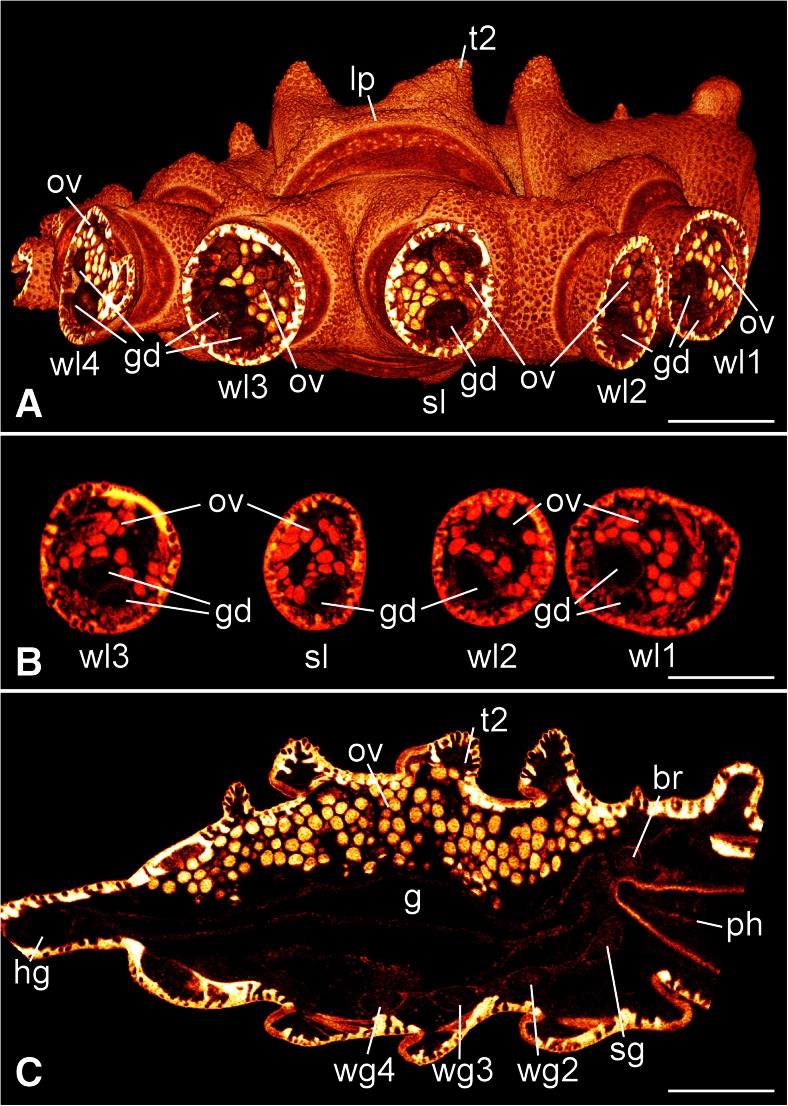
Sagittal views on the internal organization. Micro-CT scans reconstructed with Imaris 3D software. Anterior to the right. *Scale bars* 1 mm. **a** Lateral perspective on the trunk and the walking legs (*wl1–wl4*), including the supernumerary leg (*sl*), of the right body half. The large round fused structure of the lateral processes (*lp*) of the second and third walking leg segments and the slightly malformed dorsal tubercle of second walking leg segment (*t2*) are visible. The coxae 2 (first walking leg coxa 1) of the walking legs are shown as transverse sections, displaying internal structures such as gut diverticula (*gd*) and dorsal to them branches of the ovaries (*ov*). **b** Transverse optical sections of coxae 1 of the first walking leg (*wl1*) and coxae 2 of the second and third walking legs (*wl2*, *wl3*) and the supernumerary leg (*sl*). The first and the third walking legs contain two branches of gut diverticula (*gd*), whereas the second and the supernumerary walking legs possess only one diverticulum each. The branches of the ovaries (*ov*) lie above the gut diverticula. The diameter of the supernumerary leg is slightly smaller than that of the other legs. **c** Medio-sagittal optical section through the trunk posterior to the proboscis. The ovaries, the gut, and the central nervous system with the brain (*br*), the subesophageal ganglion (*sg*) including the neuromere of the first walking leg segment, and the ganglia of the second to fourth walking leg segments (*wg2*–*wg4*) are visible. *g* central midgut tube, *hg* hindgut in the anal tubercle, *ph* pharynx, *t2* deformed tubercle of the second walking leg segment

**Fig. 4:**
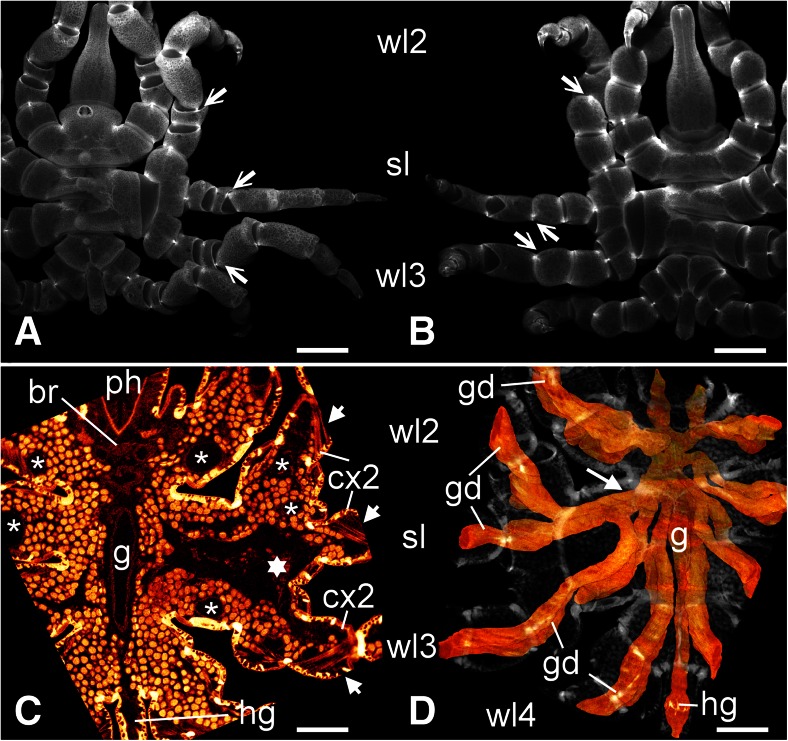
Dorsal and ventral aspects of the external and internal organization. Autofluorescence of the cuticle (**a**, **b**) and micro-CT images reconstructed with Imaris 3D software (**c**, **d**). Anterior to the top. *Scale bars* 2 mm (**a**, **b**) and 1 mm (**c**, **d**). **a** Dorsal view of the specimen. The trunk malformations and the mirror image of the supernumerary leg (*sl*) as indicated by the relative position of the joints can be seen. Note especially the mirror-image position of the joints between coxa 3 and femur of the supernumerary leg in comparison to those of the second and third walking legs (*wl2*, *wl3*) (*arrows*). The image serves as orientation for understanding the topology of the internal organization in **c. b** Ventral view of the specimen. The trunk malformations and the mirror image of the supernumerary leg (*sl*) as indicated by the relative position of the joints can be seen. Note especially the mirror-image position of the joints between coxa 3 and femur of the supernumerary leg in comparison to those of the second and third walking legs (*wl2*, *wl3*) (*arrows*). The image serves as orientation for understanding the topology of the internal organization in **d**. *wl2*, *wl3* second and third walking legs. **c** Horizontal optical section of the trunk at a subdorsal level. The deviating arrangement of the ovaries (*ov*) in the fused lateral process and the coxae 1 of the second walking leg (*wl2*), the supernumerary leg (*sl*) and the third walking leg (*wl3*) is recognizable in the right body half. Note the conspicuous dorsal area that lacks proper tissue (*star*). The normal pattern is seen in the left body half. The dorsal protrusions of the gut diverticula are seen as round structures penetrating the ovaries (*asterisks*). The pattern of intrinsic leg muscles in the coxae 2 (*cx2*) reveals the mirror image symmetry of the internal structures of the supernumerary leg (*short arrows*). For orientation, compare with **a. d** Ventral aspect of the 3D reconstruction of the gut diverticula (*gd*). Gut orange, external structures, and ovaries *transparent gray*. The branching pattern of gut diverticula that lead into the legs is different between the normal right body half and the malformed body half (see text). In the latter, note the in part common origin and the connections between the second and third walking leg diverticula and that of the supernumerary leg (*long arrow*). For orientation, compare with **b**. *br* brain, *g* central midgut tube, *hg* hindgut in the anal tubercle, *ph* pharynx

The malformed specimen shows a normal left body half (Fig. 1). On the right side, the anterior and the posterior body parts are also normally expressed (Fig. 1) and the lesion concerns only the segments of the right second and third walking legs (Figs. 1 and 2). The trunk in this area is characterized by a somewhat distorted pattern in the dorsal midline and an oblique ventral segmental furrow. Most strikingly, however, it shows a fused right half in which the lateral processes of the second and third walking legs form one large unit that is round in transverse section and shows no visible segmental boundary (Figs. 1, 2, 3a, and 4a, b). Three legs originate from this large fused lateral process. These are in an anterior-posterior sequence: the second walking leg, the supernumerary leg, and the third walking leg (Figs. 1, 2, 3a, and 4a, b). The coxae 1 of these three legs are distinct structures. Nevertheless, they are proximally fused, and those of the second walking leg and of the supernumerary leg show a higher degree of fusion than that of the latter to the coxa 1 of the third walking leg (Figs. 1, 2a, b, and 4a, b). Beginning with coxa 2, all three legs are normally developed with respect to podomere shapes and numbers (Figs. 1, 2c, and 4a, b), i.e., they are comprised of coxae 2 and 3, femur, tibiae 1 and 2, tarsus, propodus, and terminal claw as is characteristic for pycnogonid walking legs (Fig. 2c). Yet, the supernumerary leg is slightly smaller (Figs. 1 and 2c) and shows a mirror image organization, which is evident from the position of the articulations between the podomeres and the arrangement of intrinsic limb muscles (Fig. 4a–c). Especially the articulation between coxa 3 and femur, the plane of movement created by its two joints showing a 45° rotation with respect to the anterior-posterior as well as dorsal-ventral body axes (see Vilpoux and Waloszek 2003), illustrates unequivocally the mirror image organization (Figs. 2c and 4a, b).

#### Internal organization

A derived feature of pycnogonids is that internal organs such as gut diverticula and gonads are extending into the legs (Arnaud and Bamber 1987). This is also true for the malformed specimen described here. Yet, the internal body organization in the region of the lesion has also been affected by morphological changes (Figs. 3 and 4c, d). In particular, the gut and the ovaries show differences to their normal morphology.

In the unharmed body half, the ovaries occupy the space lateral and dorsal to the gut throughout the trunk with its segmental lateral processes (Figs. 3c and 4c, d). Furthermore, they send segmental extensions via the lateral processes into the four walking legs. This situation corresponds well to earlier descriptions of the internal anatomy of *P. litorale* (Helfer and Schlottke 1935). In contrast to this, the area of the fused right lateral processes and the fused coxae 1 comprises a relatively large dorsal area in which no part of the ovary or oocytes can be found (Fig. 4c). Instead, there is an open space that is only loosely filled with unspecified connective tissue. This open space is ventrally and laterally enclosed by an undivided ovary sheet from which extensions separate at the level of the coxae 2 and lead into the second and third walking limbs and into the supernumerary leg (Figs. 3a, b and 4c).

In the unharmed body half, a single gut diverticulum separates from the central longitudinal midgut for each segmental walking leg (Fig. 4d). In accordance with earlier descriptions, each diverticulum then bifurcates within the trunk into a dorsal and a ventral branch, both of which continuing into the respective leg (Helfer and Schlottke 1935). The dorsal branch, in particular, shows some short bulges mainly in dorsal direction, which protrude between the filled ovaries (Fig. 4c). The malformed parts of the injured right body half reveal some deviations from this general pattern (Fig. 4d). The roots of the second and third lateral gut diverticula are not entirely separated. Furthermore, the diverticulum that leads into the supernumerary limb branches off from the diverticulum of the second walking leg, and there is an additional connection to the ventral branch of the third walking leg (Fig. 4d). While the latter limb shows the normal pattern of a dorsal and a ventral gut diverticulum in its more distal regions, the second walking leg and the supernumerary leg each contain only a single diverticulum (Fig. 3a, b).

The nervous system could not be studied in great detail due to its relatively low contrast and resolution in the micro-CT data. Nevertheless, number, gross shape, and position of the segmental ganglia are unaffected and correspond to the normal situation in the ventral nerve cord of *P. litorale* (e.g., Brenneis and Scholtz 2015) (Fig. 3c). Neither the segmental nerve of the second walking leg nor a nerve targeting the supernumerary leg was traceable in the scan. However, the segmental nerve of the third walking leg could be traced and shows no branch projecting into the supernumerary limb. Based on this and on the fact that the supernumerary leg was moving when the animal was alive, its innervation can be inferred—most likely from the second walking leg ganglion.

The arrangement of musculature of the proximal podomeres (especially coxa 2) confirms that the supernumerary leg has a mirror image orientation compared with the other limbs of the right body half (Fig. 4c).

## Discussion

### Regeneration in Pycnogonida and the connection between leg structures and internal organs

Regeneration is a complex process and may be affected by various perturbations that in the end may lead to malformed structures (e.g., Korschelt 1907; Przibram 1909, 1921; Needham 1952; Charmantier-Daures and Vernet 2004; Maruzzo et al. 2005). Such malformations are of interest because they can contribute to the understanding of developmental mechanisms. Moreover, they show what is morphologically possible; they reveal a morphological potential, a surplus that is not expressed under normal circumstances.

Like other arthropods, Pycnogonida show a certain ability of regeneration (Dohrn 1881; Loeb 1895; Gaubert 1892; Helfer and Schlottke 1935; Hedgpeth 1947). Ablation experiments of trunk segments and limbs led to an at least partial regeneration of ablated structures (Loeb 1895; Morgan 1904). Furthermore, autotomy of limbs (appendotomy sensu Maruzzo et al. 2005) has been demonstrated by several authors (Dohrn 1881; Gaubert 1892; Helfer and Schlottke 1935).

There are a number of reports on malformations in pycnogonids, which most likely resulted from irregular regeneration. Most of these reports are anecdotal descriptions of aberrations, such as missing or additional trunk and limb structures that were found among animals collected in the wild or in laboratory cultures (Dogiel 1911; Schimkewitsch and Dogiel 1913; Bouvier 1914; Gordon 1932; Arita 1936; Ohshima 1942a, b; Hedgpeth 1947; Child 1979; Stock 1987). In addition, the ablation experiments of Loeb (1895) and Morgan (1904) resulted in malformed specimens.

As mentioned above, pycnogonids are the only arthropods, in which internal organs, such as gut diverticula and gonads extend into the legs (Arnaud and Bamber 1987). This raises the question to what degree the formation of these extensions is connected to the limb formation process. The supernumerary leg of the *P. litorale* specimen studied by us is equipped with one gut diverticulum and a process of the ovary. This suggests a tight connection between the formation of typical arthropod leg structures such as muscles, nerves, and joints and the internal organs of pycnogonids. The observation that malformed pycnogonid walking legs with bifurcations or trifurcations of distal leg parts also contain at least gut diverticula in these multiplied parts point to the same direction (Schimkewitsch and Dogiel 1913; Ohshima 1942b).

The only two other reported instances of complete additional legs (Arita 1936) show contradictory patterns in this respect. Arita described a juvenile and an adult specimen of *Nymphonella tapetis* Ohshima, 1927 with a corresponding pattern of a supernumerary leg posterior to the right fourth walking limb. The extra leg of the juvenile was equipped with a projection of the gut. The supernumerary leg of the adult specimen shows the normal set of podomeres, but it is smaller than the regular walking legs. However, in contrast to the juvenile specimen, a gut diverticulum is absent. Arita (1936) explained this with the reduced size of the supernumerary leg. Whether or not this latter example falsifies the idea of a developmental integration between limb structures and the extension of internal organs remains to be tested.

### What caused the supernumerary walking leg?

Understanding malformations, if these are not experimentally produced, has to follow a backward argumentation (see Scholtz et al. 2014, p. 168) starting with the pattern of the malformed structure in order to reconstruct a plausible narrative of a scenario that may have led to the observed result. The epistemic framework for this approach has been developed by the art historian Carlo Ginzburg, who revealed a methodological tradition from Stone Age hunters via Morelli, Freud, and Sherlock Holmes in the use of clues as results of processes that can be reconstructed by interpreting these clues—be it animal tracks, painting styles, psychoses, or the outcome of a crime (see Ginzburg and Davin 1980). Although developed in the realm of the humanities, there are many instances for this epistemic approach in biology. These concern phylogenetic analyses, the interpretation of fossils, the reconstruction of scenarios for the evolution of morphological structures (e.g., the head problem in vertebrates and arthropods see Scholtz and Edgecombe 2006, p. 396), and, as mentioned above, the analysis of malformations to name a few.

In the case of a supernumerary leg in the *P. litorale* specimen presented here, some aspects of the history of the lesion are known, such as the injury in the six-legged sixth postembryonic instar between the second and the third walking legs. However, details such as the exact degree of the injury were not studied, and the further development of the individual has not been followed until it was recovered as an adult. Hence, to understand the causes and the nature of the observed malformation, it is necessary to reconstruct the aspects of its formation based on the clues from the resulting pattern. This pattern is characterized by the following features: (i) a fused lateral process with a perturbed organization of gut diverticula and ovary structures; (ii) compound coxae 1 of the second and third walking legs and of the supernumerary leg that lies between them, with a higher degree of fusion between the latter and the second walking leg; (iii) absence of a nervous connection between the supernumerary leg and the ganglion of the third walking limb segment; and (iv) an external and internal mirror image symmetry of the supernumerary leg, as revealed by the arrangement of joints and intrinsic muscles, respectively.

The fusion of the second and third lateral processes and the perturbation of the gut diverticula and ovaries indicate that the lesion affected the right side of the trunk region to a relatively large degree. In addition, the pattern of the compounded coxae 1 suggest that the proximal parts of the coxae 1 of the second and third walking legs were injured and that the supernumerary leg grew out between these two normal legs with a greater spatial affinity to the second walking leg. This is evidenced by the position and degree of fusion of the coxae 1 and the branching pattern of the gut diverticula. The absence of a nervous connection to the third walking leg ganglion points to the same direction. The closer connection between the supernumerary leg and the second walking leg might be due to a larger damage in the proximal region of the latter compared to that of the third walking leg. The missing second gut diverticulum in the second and the supernumerary walking legs may also be explained by a large lesion in the proximal parts of the second leg. Dohrn (1881, p. 81) described that during autotomy, the gut diverticula contract at a predetermined breaking point to avoid a loss of nutrients and body liquid. A process like this could have resulted in a resorption of the distal part of the diverticula in the second walking leg. In parallel to the de novo formation of the supernumerary leg with its single gut diverticulum, the diverticulum of the second walking leg would then have been regenerated, although likewise in a less complex state.

**Fig. 5:**
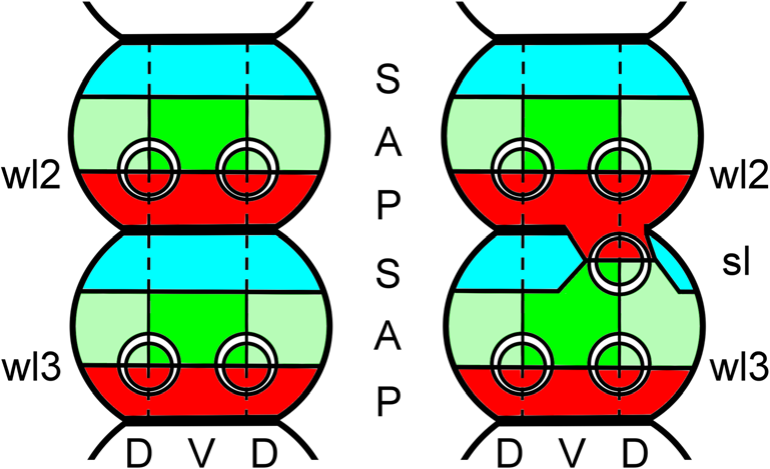
Schematic representation of the application of the boundary model to the supernumerary leg in the pycnogonid specimen. After Meinhardt (2008), modified and adapted. *Left*: The normal expression of the segments of the second and third walking legs (*wl2*, *wl3*) is shown. The three stripes of cell states in each segment are indicated by the colors *turquois* (*S*), *green* (anterior (*A*)) and *red* (posterior (*P*)). The vertical lines indicate the boundary between dorsal (*D*) and ventral (*V*) cell states. The anterior-dorsal cell state is indicated by *light green*. The limb buds (depicted by *two non-concentric circles* indicating an anterior-posterior polarity) form in the area of the boundaries between the anterior/ventral (*green*), anterior/dorsal (*light green*), and the posterior (*red*) cell states. *Right*: The supernumerary leg (*sl*) is formed after a lesion between the second and third walking leg segments. The lesion led to a loss of cells of the S cell state (*turquois*). With tissue regeneration, the ventral and dorsal A (*green/light green*) and P cell (*red*) stages get in contact forming a new boundary resulting in an supernumerary leg with reversed polarity

Unintentionally, some sort of an “experiment” has been conducted by us that resembles extirpation studies in insect segments (Bohn 1974). Bohn could show that a supernumerary leg with a mirror image symmetry is formed between two adjacent segments of the cockroach if the “leg-inducing membrane” (sensu Bohn 1974) is brought into contact with the sclerite of an adjacent segment by removing the membrane that normally separates these structures (Bohn 1974). In the 1980s, Meinhardt developed the boundary model that is able to explain Bohn’s experiments and which has been confirmed later by molecular data (Meinhardt 1986, 2008). According to this boundary model, limbs and other lateral branches of the body axis are formed at a boundary where cell populations with at least three different states meet (Fig. 5). From *Drosophila* research, it was known that each segment comprises transverse cell populations with an anterior and a posterior fate, the compartments, which lie strictly separated but adjacent to each other (Martinez Arias and Lawrence 1985). In addition, the model implies that there is a longitudinal boundary separating dorsal and ventral cells on either lateral side of the embryo (Meinhardt 1986, 2008). In the contact zone between anterior and posterior cells and the dorso-ventral border, the formation of limb buds is initiated (Fig. 5). Meinhardt’s model has been corroborated twofold: by clonal studies of crustacean segmentation and limb differentiation and by molecular genetic investigations on *Drosophila* limb development. Clonal studies on limb development in crustaceans showed that each leg is composed of cells from two adjacent genealogical units or parasegments (Dohle and Scholtz 1988; Wolff and Scholtz 2008). The early limb buds are formed at a clonal boundary (Hejnol and Scholtz 2004) and in a distinct distance to the midline which plays a central role for dorso-ventral patterning by secreting morphogenetic proteins (Vargas-Vila et al. 2010). *Drosophila* studies revealed that the anterior and posterior cell populations express different segment polarity genes such a *wingless* and *hedgehog/engrailed*, whereas the dorso-ventral boundary is marked by the *decapentaplegic* gene. At the intersection of these genes, the homeobox gene *Distal-less* is activated that initiates the budding of limbs (Campbell and Tomlinson 1995). Similar processes were shown to act during the regeneration of cricket legs (Mito et al. 2002). Although other arthropod species show a somewhat different mix of the molecular toolkits during early limb formation (see Prpic and Damen 2008), this does not necessarily mean that the model is wrong at the level of cell states. Meinhardt’s model implies a third segment polarity cell state that separates the posterior cells from the anterior cells of the next following segment (Fig. 5). Otherwise, legs with mirror image orientation would form at every posterior-anterior boundary, as was shown in the experiments of Bohn (1974). If the cells with the third cell state between adjacent segments are removed, the posterior cells of the anterior segment form a contact zone with the anterior cells of the more posterior segment and an additional mirror image leg is formed (see Meinhardt 1986, 2008). Again, Meinhardt’s implication finds support from gene expression data. In each normally developing segment anlage, there is always an anterior region of cells that do neither express *engrailed* nor *wingless* (e.g., Damen 2002).

Hence, with its supernumerary walking leg with mirror image symmetry in the region of a former intersegmental lesion, the pattern of malformation in the *P. litorale* specimen studied is in good accordance with Bohn’s experiments and Meinhardt’s boundary model.
